# Hospital and Institutional News

**Published:** 1917-07-28

**Authors:** 


### HOSPITAL AND INSTITUTIONAL NEWS.

### THE PRINCE OF WALES' FUND: REFORM IT, OR END IT.

At a recent meeting of the West Derby Board of Guardians, with Mr. P. Leonard in the chair, Mr. S. Reeves asked why it was that letters were constantly received from dependants of sailors and soldiers for assistance, and that these communications were accompanied by papers from the Prince of Wales' Fund. This procedure suggested that the Fund was simply an organisation for giving to applicants letters to Guardians asking for relief. In response to his inquiry whether this was the fact, the Clerk bluntly replied that the Fund with the separation allowances should be sufficient for a man and his family without recourse to the Poor Law. He actually had on his table a letter from the Fund asking the Guardians to assist a man and his family because the separation allowance was insufficient for their maintenance, and the Fund was "so hidebound by rules" that it was prevented from doing its duty. Every honest man and woman must regard this attitude of the Fund as a disgrace to it and its directors. If the Fund is not to assist cases of this class, what was it established to do? Nothing could damn it more than its persistent wrong-doing, in passing on soldiers' dependants to the Poor Law. It is a scandalous indictment of its own methods, and, with something like half its funds unspent, a public inquiry is urgently demanded, in the interests of our soldiers, their dependants, and others, to bring those in charge to a proper sense of their duty, or else to dismiss them as incompetent to perform it. Will some independent member of Parliament devote himself to secure the speedy removal of an evil fostered, as The Hospital has many times demonstrated, by the Prince of Wales' National Relief Fund from its establishment to the present time?

### ANOTHER LIGHT ON MESOPOTAMIA.

Speaking at the annual meeting of the Kensington Division of the British Red Cross Society, Mr. E. A. Ridsdale, vice-chairman of the executive committee of the society, explained how the Red Cross had met with a rebuff in seeking to aid the wounded in Mesopotamia. Mr. Ridsdale, who was the third member of the Vincent-Bingley Commission, which was appointed to report on the breakdown of the Medical Services, said that on July 17, 1915, Sir William Garstin, head of the stores department of the Red Cross Joint Committee, wired to General Sir John Nixon, offering to help in any way, and almost at once provided two petrol motor launches, which he undertook to equip with practical engineers. To the offer of more boats no answer was received, but the Committee nevertheless dispatched further boats which did excellent service. Finally, to a request that the Red Cross might assist in any other way, came, on December 28, 1915, the surprising reply: "Many thanks for your wire of the 16th. Nothing is required at present. If anything is needed in future, I will not hesitate to ask you." To give direct help to the fighting men would be a breach of the Geneva Convention. The Joint Committee had sent out many thousands of pounds' worth of stores before the breakdown at Ctesiphon. He (Mr. Ridsdale) went out again with Lord Chelmsford. They encountered some opposition, a great dislike to receive an emissary from this country being shown. He was welcomed by a wire from Lord Hardinge at Aden, and in the middle of April arrived at Basra with £20,000 worth of stores and some *personnel*. This speech comes as a significant postscript to the Mesopotamia Report, and proves again that the medical problem was never faced, and that those in authority seemed to be wholly ignorant of what was required and what was being done or left undone.

### A SOLDIER PATIENT'S INVENTION.

When Lord French fulfilled his first public engagement in Belfast by visiting the Ulster Volunteer Force Hospital the other day, his inspection included a remarkable novelty. At the orthopædic branch of the institution, to which Captain Metcalf, of the United States Army, is attached so as to gain experience for the establishment of similar departments in America, Lord French was privileged to see an example of the carpentry work which has been accomplished by Private Rooney, of the 3rd Battalion Royal Irish Rifles. Private Rooney lost one of his hands on active service, and when he returned to England was fitted with an artificial hand in its place. Not finding this mechanical hand as convenient as he had hoped, Private Rooney tried to improve upon it. As a result of his experiments, he has invented an appliance by which he can grasp a hammer and manipulate a saw, and, in fact, use all the tools of a carpenter. He thereupon applied for a patent for his invention, and we understand that the Pensions Committee in London has offered to purchase it. The Pensions Committee, which has not earned a famous reputation for generosity, has here an opportunity of doing so, and, for the honour of itself and the country, we hope they will not let it slip.

### DR. KEATS' PENSION.

At a recent meeting of the Camberwell Board of Guardians Mr. J. H. Cuthbert proposed that his colleagues should request the Local Government Board to hold an inquiry into the circumstances of Dr. W. J. C. Keats' resignation. It will be recalled that Dr. Keats was formerly medical superintendent of the Camberwell Infirmary, and on his resignation a superannuation allowance of £369 3s. 7d. was granted to him. This also Mr. Cuthbert wished to have brought into the scope of his proposed inquiry. He contended that the sole ground for a grant of a superannuation allowance was the permanent mental and bodily incapacity of the doctor. He questioned whether this had occurred, and asked whether, if so, the work had been too strenuous or whether something among his staff had unnerved him. In spite of three medical certificates declaring Dr. Keats to be incapable of supporting his duties, within six months of leaving the infirmary he was living with Dr. Hickman at The Hollies, Wanstead, and since the latter's death had been carrying on his practice. The clerk produced the three medical certificates relating to Dr. Keats' condition in June last year. All of them testified that he was suffering from a mental and nervous breakdown, and that he was permanently incapacitated from carrying on his duties under the board. In two it was stated that a period of rest might enable him to undertake light work. Mr. Cuthbert's proposal was defeated by a large majority. The Guardians properly took the view that Dr. Keats' present responsibilities could not be compared with the heavy weight of administration in the infirmary.

### THE LONDON HOSPITAL AND AIR-RAIDS.

The following is the text of a notice, printed in English and Yiddish, outside the London Hospital. It sufficiently explains itself: "We have hitherto allowed people who might happen to be in the open in Whitechapel Road when an air-raid warning has been received to take cover in the London Hospital. On account of the behaviour of the crowd on Saturday last, July 14, we shall do so no more. The crowd, consisting almost entirely of foreigners, a large number of whom were healthy young men, came in hundreds, leaving their houses, and bringing whole families with them. By their shouting and screaming sick patients were seriously alarmed, and the engineers were prevented from getting to their posts in case of fire. It would have been impossible to admit wounded had it been necessary. After this experience we shall lock up all entrances to the hospital, and none but wounded and injured will be allowed to enter." Other East-end hospitals are for the same reason considering the same course, which has the approval of the police.

### ATTEMPT TO BRIBE A HALL PORTER.

The following letter, which has been published in *Truth*, deserves to be placed on record as a typical attempt of a solicitor's tout to corrupt the hall porter of a hospital. It was addressed to the hall porter of Guy's Hospital by Mr. Guy R. Preston, 66 Great Portland Street, Oxford Street, W., who, describing himself as "claims assessor," is also to be found at 75 Highgate Hill, N. The letter, which is marked "Private and confidential," runs:—

Dear Sir,—It has occurred to me that, in your capacity as attendant to your hospital, all cases of street and other accidents (excepting always those caused by hostile aircraft) come under your personal notice before entering the institution, and as I am desirous in such cases of communicating with the injured at the earliest possible moment after their admittance to the respective wards, I shall be pleased if you will at once wire or 'phone me full particulars of any such cases, and I will at all times *immediately* refund any expenses incurred, and in addition thereto I am prepared to present you with my cheque for two guineas upon every case taken up by me together with a further payment of 10 per cent. upon any profits I may derive from damages awarded to the injured, and which will, of course, enable you to substantially add to your income. I shall further, at all times, respect your agency as *entirely confidential*, and think the same should turn out an extremely profitable one in view of the many such cases admitted to your hospital and of your being my *sole* agent here, in which case you will do well to look after my interests.

Your early reply will oblige.—Yours faithfully,

Guy R. Preston.

As *Truth* aptly remarks, the method by which solicitors' clerks get in touch so promptly with the victim of an accident or his relatives is now revealed. What is the Law Society doing in this matter?

### A PHYSICIAN BENEFITS HIS HOSPITAL.

The death of Dr. James Milward, at the age of seventy-eight, recalls an interesting conditional gift to his training school, Guy's Hospital. Dr. Milward had practised in Cardiff and held various appointments, and his professional skill and enthusiasm, combined with his kind and sympathetic character, made him universally beloved. In 1916, Dr. Milward was the owner of an estate between the towns of Cardiff and Barry Docks. The land, twenty-four acres, was built over, and the houses, let on long leases, produced a rental of £1,035. The estate was, however, subject to a mortgage of £12,250, and with this encumbrance Dr. Milward, last year, arranged to make over the whole property to Guy's Hospital on condition that an annuity of £300 should be paid to the doctor and his wife during their lives and the life of the survivor. The immediate value of the gift was of course not great, but the potential value was considerable, and the estate will no doubt prove, in years to come, a source of considerable revenue. A portion of the estate is still undeveloped, and as Barry Docks are becoming increasingly important there is an excellent chance of land in the neighbourhood becoming very valuable. Some final business in connection with the transaction had been arranged for noon on June 19, when Dr. Milward had to meet the hospital's solicitors, but he died suddenly during the night of June 18.

### AID FOR WOMEN MEDICAL STUDENTS.

The General Committee for the Promotion of the Medical Training of Women held its annual meeting on July 17 at the University College of North Wales, Bangor. The General Committee was established at a meeting in February 1916 held at the University College, Cardiff, with the object of enabling a larger number of women to enter the medical profession, as it was felt that the war conditions prevailing were increasing the demand for medical services, while at the same time there were heavy losses among the medical men serving abroad and, further, many medical students were relinquishing their studies to serve as combatants. It was explained that the organisation was intended to serve the whole of Wales, assisting students from all parts of the Principality. One speaker held that it was very important that grants in aid should not be given till the fourth year of studentship, and that no grant should be given except in absolutely justifiable cases. By deferring the grants to the later years of the student's curriculum the committee was able to assist those students who had done well and were persevering. The Vice-Chancellor of the University of Wales, Principal E. H. Griffith, maintained that the secondary schools did not give the education required, for the girls entering as medical students did not possess a good knowledge of mathematics, and this made it much harder for them to study science when they came to the colleges. The committee was stated to possess a credit balance of £394, but against this there were several liabilities. The movement is important, and it will be of interest to see what real effect it has on the supply of women medical students.

### THE FOOD REQUIRED BY THE NATION.

In a recent lecture Professor Noel Paton, Professor of Physiology in the University of Glasgow, gave the results of some interesting calculations on the minimum amount of food needed for the nourishment of the whole country. He started from the ascertained fact that a man doing moderate work should take daily food containing 3,400 units of energy or "calories." This amount has been based on a large number of men, and checked by the examination of the diets of men under various conditions, and with varying amounts of work, so that the result may be taken as at least fairly accurate. Next the requirements of women and children of different ages have been calculated as definite fractions of those of a man doing moderate work. By applying these data to the numbers given in the Census return of 1911, it is found that the whole population was nearly equivalent to thirty-five million "men" and, therefore, it is clear that if these facts are reliable the total amount of food required for the whole country must amount to 43½ millions of millions of calories per annum. A study of the food supply of the nation in the days before the war shows that a considerable excess was consumed, the total being over 4,000 calories per "man" per day. As to the source of these units of energy it has been found that cereals supply about 35 per cent., meat about 27 per cent., milk and butter about 18 per cent., potatoes about 8 per cent., and all other foodstuffs make up the remaining 12 per cent.

### LIVERPOOL'S HOSPITAL SATURDAY FUND.

Liverpool has reason to congratulate itself upon the fact that, although appeals to its charitably disposed citizens were never so numerous as in these days of war, the people of the city have shown their determination not to let the hospitals suffer. The working men especially have risen to the occasion splendidly, the collections made in the workshops on behalf of the Hospital Saturday Fund showing an increase of £650 this year. There is a substantial advance also in the ladies' street collection, so that, in all, the Fund will exceed last year's amount by more than £1,000.

### A PROGRESSIVE INSTITUTION.

Interesting details regarding the development of the Carnarvonshire and Anglesey Infirmary, Bangor, were forthcoming last week, when Lord Colwyn opened a new temporary ward which has been erected in the grounds for the purpose of relieving the increasing pressure on the main buildings. This extension, which is an airy and well-lighted wooden structure containing twenty beds, has cost £500. Mr. J. R. Davies, chairman of the governors, pointed out that the infirmary was overcrowded, and that a complete rebuilding of the institution was desirable. The buildings had been erected many years ago, and were most inconvenient and out of date. Dr. E. O. Price, speaking on behalf of the staff, thanked the governors for falling in with their views, and pointed out that the infirmary was likely to be a clinical centre for a large portion of North Wales. He stated that a public health laboratory was to be established there in connection with the University College of North Wales, and the institution was to be utilised for a maternity scheme for poor women and the care of infants and children, and also for the treatment of 50,000 insured persons in the two counties. "Provision is also required," he added, "for the middle class—the backbone of the country. The rich and the poor get every attention, but the middle class cannot afford to pay big fees. They are left out in the cold." Dr. J. E. Thomas spoke of the ever-increasing demands on the infirmary, which is now dealing with two patients for every one treated five years ago. In 1906 only fifty-seven operations, most of a minor character, were performed; in 1916 the number had jumped to 243. During the last four years the infirmary has been without a house surgeon. The new ward, said Dr. Thomas, would be totally inadequate to meet the minimum demands in three years' time, and he looked forward to a wing being added at a cost of about £20,000, which would give them another eighty beds at least.

### THE PERSONAL SERVICE TRADITION.

It is the happy fortune of the Providence Free Hospital, St. Helens, to be able to fill the great gap caused by the recent death of Sir Joseph B. Leach, who for many years had been its chairman, by electing to the vacant seat his son, Mr. W. H. Leach, J.P. Seldom are the circumstances so fortunate, and there can be no surer guarantee of a continuity of keen interest and an absorbing desire for the institution's progress and development than this following of the son in the footsteps of his father. The hospital recently lost another good friend in Sir Joseph Beecham, who had been intimately connected with it for a long period as treasurer. It is a matter for congratulation that the memory of these two benefactors of the Providence Hospital is to be perpetuated in extensions made possible by the generosity of their children.

### HOLIDAYS AND HOSPITAL PATIENTS.

Sir Edward Holden's appeal to Lancastrians in London for visitors to the many Lancashire soldiers in the Metropolitan hospitals recalls the fact that the approaching holiday season makes the problem of securing the regular supply of visitors a general one. If the wounded are to be neglected it is in the holidays that they will be so, and therefore it is well to recall the existence of the County Folk Visitation Society, which looks after the visiting of men of both Services in London hospitals. The society keeps a list of the counties representatives of which are most in request. The society's address is 35 Pelham Street, South Kensington, S.W. 7.

### PRIVATE WARDS IN COTTAGE HOSPITALS.

Although at present there is a deficit of £381 on the extension of Willesden Cottage Hospital, the chairman and the vice-chairman have both expressed the view that this has been a good investment, since it includes the putting up of private wards. This testimony is one that should encourage other cottage hospitals to make similar additions, although the Willesden institution can hardly be considered a cottage hospital. At present there are fifty-three patients and a total of sixty-three beds, of which forty-one are occupied by soldiers. In the recreation hut eight men have been accommodated. This is a sign of growth which should be appreciated in the district, and make the clearing off of the deficit an easy matter. Willesden is exactly the type of locality where private wards should be in constant request, and where nothing else exists which can replace them.

### HOSPITAL WATER SUPPLY.

The Metropolitan Water Board, which has to levy a deficiency rate every year to make good its losses, has been looking around to see if it is possible to secure additional revenue, and finds that almost the only way would be to withdraw the concessions now granted to hospitals. These institutions are entitled to be supplied at the ordinary domestic rate assessed on the rateable value, providing (*a*) they are wholly or partly supported by endowments or voluntary contributions and not carried on for private profit or gain, and (*b*) their fittings comply with the regulations of the Water Board. In many cases such compliance as to regulations is absent, although the consumption is heavy. Hitherto the Board has been indulgent with regard to these hospital fittings, but, if it chose to change its attitude, it could enforce a meter supply so long as the fittings remained irregular. Upon this point a special commitee of the Water Board has been inquiring into the matter, and it reports that the provision of regulation fittings would be a matter of great cost and a serious impediment to the work of the institutions. Hence the committee concludes that it is probable that there would be an outcry if the higher charges were imposed. A further point referred to by the committee is that the rating of hospitals is very low, and that some additional revenue could be obtained if the Board felt that it would be right to enforce an increase in these values. The Water Board, fortunately, is doing nothing with regard to the possibility of increasing its revenue from the hospitals, which, for the time being at all events, will continue to enjoy immunity from the full water-rates which the Water Board might be able to enforce should it feel so disposed.

### TWENTY-NINE YEARS' SERVICE.

Mr. Arthur S. Mather has been appointed a vice-president of the Woolton Convalescent Institution, of which he has been a member of the council for twenty-nine years. Mr. Mather only gave up his position as chairman during the year in which he held the post of Lord Mayor of Liverpool. This recognition of his long period of service has, therefore, been well earned.

### POOR-LAW OFFICERS AND A MINISTRY OF HEALTH.

At the recent summer conference of the Gloucestershire and Somerset branch of the National Poor-Law Officers' Association a resolution was passed which "viewed with great concern any scheme which would remove the medical treatment of the poor from the direct control of the Guardians." The resolution, which was inspired by nervousness at the suggested creation of a Ministry of Public Health, urged the national executive of the Association "to safeguard the interests of the Poor-Law Service." We quote it as a relic of the old tradition, for whether such a Ministry be created or not, the first and essential reform in the medical treatment of the poor is to separate the Poor-Law infirmaries wholly from the workhouse influence and control. This, in fact, is bound to come, even in the case of the so-called sick-wards of the workhouses.

### GUY'S DENTAL SURGEONS AND MILITARY SERVICE.

Dr. M. S. Pembrey had again to appear before the Southwark Tribunal for the exemption of a third dental house surgeon for Guy's Hospital, but unfortunately on this occasion he was not able to satisfy the Tribunal as to the great need there is to retain a third house surgeon at the hospital. The Tribunal decided that the house surgeon should have a month's final exemption, and Dr. Pembrey at once notified his intention of appealing against the decision, and, judging by the result of other cases which have been taken to the Appeal Tribunal for final decision by the Military Representative, he should have little difficulty in once again convincing those sitting at Spring Gardens of the needs of the London poor and the necessity of retaining at least a modified dental service for them at Guy's. Dr. Pembrey took the opportunity of refuting the aspersion cast upon the dental students at Guy's, which was referred to in the last issue of The Hospital, when the Military Representative said that in order to avoid military service a man should have become a dental student at Guy's in 1914, by pointing out that those for whom he had appealed were students at the hospital in 1913, before the outbreak of war. It is most unfortunate in the interests of the hospital and of the military authorities that there should be any dispute of this kind, but there is little doubt that the Appeal Tribunal will see that justice is done to everyone associated with the work of a great hospital such as Guy's. The simple question is whether it is better in the interests of the hospital, the dental department, and the thousands of poor not only from the south side of the Thames, but from all over London, that three dental house surgeons should be retained, or whether they should be sent into the fighting ranks of the Army. The Military Representative at Southwark informed the Tribunal that if the dental surgeons had to join up their dental experiences would not be taken into account, but they would have to go into the fighting-line.

### THE NEED FOR MORE SANATORIUM BEDS.

THE problem of provision of sanatorium beds gets on slowly. It was by no means settled before the war; and that event has, of course, practically stopped building, while at the same time it increased enormously the number of patients. In Austro-Hungary, especially in the Bukowina and in Bosnia, tuberculosis has made great ravages, so that a conference of Austrian physicians has recommended the erection of sanatoria composed of wooden huts in order to cope with the disease quickly. In our own country, however, in spite of the fewer opportunities of consultation owing to shortage of staff, and in spite of the increased cost of travelling, patients' attendances at tuberculosis dispensaries have gone up considerably. The sanatorium waiting-lists have lengthened, and in some, probably many, districts a consumptive is lucky to get admitted now under three months after being recommended. The question has been lately raised by District Councils in Cumberland (where the Meathop Sanatorium is a gratifying instance of what can be done by energy and good management without the backing of a large population) and in Doncaster; for, as has been stated, every day's, or at any rate week's delay in the matter militates against the patient's ultimate recovery. The tuberculosis problem was, of course, always at bottom an economic one. At the present time, every expenditure of the national resources has to be planned on that basis. The suggestion is made that if it is admissible to appropriate structures like parish rooms, country houses, scantily used chapels, and so forth, to the use of the wounded, it is also admissible to modify and employ them to serve the cause of the public health.

### THE INFANT DEATH-RATE AT WARRINGTON.

BY the adoption of preventive methods the infantile death-rate at Warrington has been considerably lessened; but, with the institution of a municipal maternity home, which the Corporation decided to establish at its last meeting, it is hoped to effect a still further reduction. The medical officer of health, Dr. G. W. N. Joseph, in a report which he presented, pointed out that many of the still-births were preventable if only the mothers could have advice and treatment sufficiently early. A house, with the required accommodation and in a good situation, is available in Victoria Park, and the immediate cost—which the chairman of the Health Committee, Alderman James Evans, was careful to point out was only the initial sum—is estimated at £450, towards which, if the scheme is approved, the Local Government Board will refund half. Once the municipal maternity home is established, it is safe to say that Warrington will extend and develop this branch of its life-saving service.

### HEALTH IN JEOPARDY.

PUBLIC health is endangered by the dearth of sanitary inspectors. Naturally these officers are difficult to obtain in these days, and some authorities are appointing ladies, who ought to be able to perform the duties with satisfaction. Evidence of the danger arising from depleted public-health staffs is given by the Medical Officer of Health of Southwark, who declares that many Houses have become foul and insanitary because there are not enough inspectors periodically to visit them. He points out that this is a serious menace to public health, and the Council, realising this, has agreed to endeavour to get more inspectors.

### WHERE ECONOMY FAILS.

AN experiment in economy tried by the L.C.C. has failed. Eighteen months ago, in order to release doctors for war service, it was arranged to examine only those school children selected by the teachers and nurses in place of the medical examination of all entrants. This scheme released the equivalent of 4½ full-time doctors and four full-time nurses. Now the Council finds the method unsatisfactory, inasmuch as there is a possibility that some children who may be suffering from hidden defects may have escaped notice. The Council, therefore, is to revert to the old method, and understands that at present there will be no difficulty in obtaining the help of qualified women practitioners and of men over military age.

### THE HEALTH OF THE CHILDREN OF SUTTON COLDFIELD.

THE report of the medical officer of health contains a report from the county health visitor, Mrs. Richardson. She stated that she had found that during the past year the health of the children had been much below the average; the babies born during the year were small, feeble, and of low vitality. She attributed this to the abnormal conditions under which the mothers were living. They suffered much from anxiety arising from the absence of their husbands, and to this was added the strain of keeping the home together in a time of greatly inflated prices. Moreover, the mother, in many cases, had to contribute to the income of the family by working, and there was also the added feeling of responsibility caused by the husband's absence. All these causes tended to lower the mother's vitality, and, therefore, the children born during the war required more care. However, the health visitor was able to report that there was already some improvement. Similar deterioration has been noticed throughout the country, and, in times like this, special care needs to be taken that the children's vitality is not allowed to become lowered.

### THE DEARTH OF DENTISTS.

When the Dental Register was started in 1878 anyone who had the slightest claim to have acted as a dentist was allowed to put his name on it, even though he held no qualification from any examining board. In the forty years which have elapsed since then very many of these have been eliminated by death, and the number of those who have become qualified since is much less than those who have died, so that it must be acknowledged that there is obviously a real scarcity in dental practitioners. The Lord President of the Council has appointed a Departmental Committee to investigate the extent and gravity of the evils connected with the practice of dentistry by persons not qualified under the Dental Acts. The Committee has to inquire into the causes of the present inadequate supply of qualified dentists, the expediency of legislation prohibiting the practice of dentistry by unqualified persons, and the advisability of permitting certain classes of those unqualified persons now practising to continue in practice. The reference to the Committee also suggests that it may be well to modify the course of study and examinations for dental students, to reduce the time and diminish the cost. The report of the Committee should prove of great interest, and we hope that it may be forthcoming in a shorter time than is usual with the reports of such Committees. Something must evidently be done to restrain the activities of the very large number of unqualified dentists now practising in this country.

### RATS AND THE PORT OF LIVERPOOL.

Dr. E. W. Hope, the Medical Officer of Health to the Port of Liverpool Sanitary Authority, has issued his annual report. In it he states that in November 1916 a sanitary survey of the port was made by inspectors from the Local Government Board, Dr. Bruce Low and Dr. Wilkinson. The preliminary inquiry was attended by officials from the Admiralty and by representatives of other authorities. The special object of the survey was to see what arrangements were necessary for preventing the introduction of disease. When hostilities come to an end and troops are brought back from the many scenes of their operations, there will be no small risk that diseases foreign to this country may be introduced, and probably the most likely of all these is plague. The connection between plague and rats has now been proved to the complete satisfaction of all those who have considered the evidence, and also it is equally certain that the rat acts through the rat flea. It is possible also that rags may be the medium through which the fleas are introduced. Therefore, it is of the highest importance that there should be no chance for rats to escape from the ships to the port. Plague has occurred during the past year at three ports in this country, at Bristol, Hull, and Liverpool. At Liverpool there have been six cases, with four deaths. The outbreak was limited to three families. In one family the man first attacked worked at a grain warehouse on the dock estate where were found infected rats. In another case a woman affected worked in a rag warehouse. In Bristol also the cases originated in a rag warehouse.

### FUMIGATION RECOMMENDED.

The Port Sanitary Authority is always on the look-out for sick or dead rats, and when one is found it is examined for the plague bacillus. Many rats also are caught and examined, and Dr. Hope explains that the killing of rats by poison is not advised, as the ship in which the poisoned rats are found is liable to be suspected of having plague rats on board. It is far better, he says, to trap the rats, as then they can be examined. During the year plague-infected rats were discovered simultaneously at two places on the dock estate, and energetic means were taken at once to minimise the risk of the extension of the disease. The fumigation of vessels whilst in port is considered the most important means of exterminating rats on shipboard, and regular fumigation is the most effectual method of keeping a ship free from rats. In the seventy ships fumigated during the year, 2,115 rats were killed. In all, 87,000 rats were killed in the port and in the city. It is essential, if any real good is to be done, that these measures should be carried out persistently, for any relaxation even for a few months would be followed by a great increase in the number of rats and in the risk of plague.

### THIS WEEK'S DRUG MARKET.

A distinctly quiet tone prevails; this is not unusual at the present season of the year in normal times, but in wartime there is seldom an uneventful week, since the constant needs of the Medical Services of our own and our Allies' armies are always needing prompt attention. It is now possible to form a more definite opinion as to the prospects of the English medicinal herb crops, and, unfortunately, this is by no means favourable on the whole. Lavender has not come up to expectations, owing to the not infrequent heavy rains; comparatively little oil of lavender is likely to be distilled. Peppermint will no doubt be a small crop. Such old-fashioned herbs as feverfew, hyssop, chamomiles, and pennyroyal are variously reported upon by different growers, but, generally speaking, the crops will probably not be much below the average. Price changes during the week have been remarkably few in number. The improved demand for quinine has been maintained and the price is again slightly higher. The advanced prices of salicylates continue, but there has been no further advance. Aspirin is unchanged in price. Phenacetin and barbitone remain scarce. Formaldehyde is rather dearer. Cream of tartar still has an advancing tendency, but citric and tartaric acids are steadier.

### TO OUR READERS.

**Contributions are specially invited from any of our readers to these columns. They should deal with topical subjects and news. They must be authenticated for the information of the Editor only. The minimum payment if published is 5s. There is no hard-and-fast rule as to space, but notes of about twenty lines in length are preferred.**

